# Ontario Veterinary College

**Published:** 1898-07

**Authors:** 


### ONTARIO VETERINARY COLLEGE.

This institution inaugurated its first course of lectures in 1862. Its first graduates in 1866 numbered three, while the total number, including the class of 1898, now reach 2275. The first building erected for instruction in veterinary science exclusively was in 1869. This building was enlarged in 1876, when the facilities would accommodate some two hundred students. In 1885 the classes had risen to over three hundred, which necessitated increased accommodations, which were completed in 1889 and now can accommodate at least five hundred students. These latter buildings have added much to the value of instruction given by providing ample laboratory facilities and rooms for class teaching.

Much importance is attached to the requirement that students shall serve an apprenticeship with graduates between the first and second-year sessions. Students are graduated in March and December of each year, which makes provision in the curriculum for students to enter in December.

Prof. Andrew Smith has been the active head of this institution from its inception to the present day. He has held many official positions in the Province of Ontario and is active in the promotion of horse-breeding, dairy development, and livestock pursuits as associated with agriculture, and has given much time and thought in the encouragement of every diverse interest of livestock industry.

The probable adoption of a compulsory three-years' course at an early date gives much promise to the college in more thoroughly educating men for the veterinary profession, and no doubt will be followed by every two-years' college advancing to the three-years' limit. Its present facilities will afford it an exceptional opportunity of giving a thorough graded course, and, with the largest number of alumni in North America to aid it, will no doubt again in the near future see its classes greatly augmented in numbers and of higher preparatory education.

